# Prevalence of hepatitis C virus infection in patients with lymphoproliferative disorders in Southern Turkey

**DOI:** 10.1038/sj.bjc.6690522

**Published:** 1999-07

**Authors:** S Paydas, B Kiliç, B Sahin, R Buğdayci

**Affiliations:** Departments of Oncology, Blood Center, Biostatistics, Çukurova University, Faculty of Medicine, Adana, Balcali, 01330, Turkey

## Abstract

Anti-hepatitis C virus (HCV) antibody prevalence was investigated in 228 patients with lymphoproliferative disorders (LPDs). Twenty-six of 228 (11.40%) patients with LPDs were positive for anti-HCV which was higher than the donor population (*P* = 0.0007). Nine of 98 cases with non-Hodgkin's lymphoma, five of 47 cases with multiple myeloma, seven of 36 cases with Hodgkin's disease, four of 38 cases with chronic lymphocytic leukaemia and one of nine cases with acute lymphoblastic leukaemia had anti-HCV antibody. In all patients, odds ratio (OR) for anti-HCV was 24.09. This value was higher in patients less than 35 years as 62.04 for below 25 years and 32.00 for between 25–35 years. Our findings suggest that HCV infection might be a causative and/or contributing factor in lymphoproliferation. © 1999 Cancer Research Campaign


*British Journal of Cancer* (1999) **80**(9), 1303–1305
© 1999 Cancer Research Campaign
Article no. bjoc.1999.0522

### Prevalence of hepatitis C virus infection in patients with lymphoproliferative disorders in Southern Turkey

**S Paydas[1], B Kiliç[2], B Sahin[1] and R Buğdayci[3]**

Çukurova University, Faculty of Medicine, Departments of [1]Oncology, [2]Blood Center and [3]Biostatistics, 01330 Balcali, Adana, Turkey

**Summary** Anti-hepatitis C virus (HCV) antibody prevalence was investigated in 228 patients with lymphoproliferative disorders (LPDs). Twenty-six of 228 (11.40%) patients with LPDs were positive for anti-HCV which was higher than the donor population ($P = 0.0007$). Nine of 98 cases with non-Hodgkin's lymphoma, five of 47 cases with multiple myeloma, seven of 36 cases with Hodgkin's disease, four of 38 cases with chronic lymphocytic leukaemia and one of nine cases with acute lymphoblastic leukaemia had anti-HCV antibody. In all patients, odds ratio (OR) for anti-HCV was 24.09. This value was higher in patients less than 35 years as 62.04 for below 25 years and 32.00 for between 25–35 years. Our findings suggest that HCV infection might be a causative and/or contributing factor in lymphoproliferation.

**Keywords:**

Chronic hepatitis C virus (HCV) infection has been associated with several extrahepatic disorders including autoimmune thyroiditis, lichen planus and essential mixed cryoglobulinaemia. These associations suggest that HCV may act as a trigger for the development of various immune-mediated disorders. HCV is a hepatotropic as well as a lymphotropic virus and can be responsible not only for chronic liver disease but also for lymphoproliferative disorders (LPDs) (Pozzato et al, 1994). As a result of its lymphotropic properties, HCV has been proposed as a possible causative factor of essential mixed cryoglobulinaemia and may trigger the monoclonal B-cell disorders such as non-Hodgkin's lymphoma (Zignego et al, 1992; Ferri et al, 1993). In this study we investigated the prevalence of HCV in LPDs and compared it to the donor population living in Southern Turkey.

### MATERIALS AND METHODS

Serum samples taken before treatment in 228 patients with LPDs were used as the study material. Non-Hodgkin's lymphoma (NHL), multiple myeloma (MM), Hodgkin's disease (HD), chronic lymphocytic leukaemia (CLL) and acute lymphoblastic leukaemia (ALL) were present in 98, 47, 36, 38 and nine patients respectively. The diagnoses were made according to diagnostic criteria including histological confirmation of biopsy materials, microscopic evaluation of peripheral blood and bone marrow samples, biochemical parameters, and radiological findings. As a control group, 36 226 donors were included in the study.

Antibodies against HCV were detected using the Third-Generation ELISA kit (Abbott). Chi-square, Spearman rank correlation, Mantel–Haenszel, Yates and Fisher's exact tests were used in order to assess the HCV risk.

*Received 10 September 1998*
*Revised 12 January 1999*
*Accepted 27 January 1999*

*Correspondence to:* S Paydas

### Findings

The study group comprised 228 cases with LPDs. Anti-HCV antibodies were positive in nine of 98 (9.18%) cases with NHL, five of 47 (10.63%) cases with MM, seven of 36 (19.44%) cases with HD, four of 38 (10.52%) cases with CLL and one of nine (11.11%) cases with ALL. Serum alanine aminotransferase levels were normal in all except two of the seropositive patients. The characteristics of the patients are shown in Table 1. Twenty-six of 228 (11.40%) patients had antibody to HCV; anti-HCV prevalence in the donor group was 0.53%. Anti-HCV antibody was found to be increased in patients with LPDs as compared to the donor population ($P = 0.00000$). A significant trend by age in anti-HCV positivity was observed in the donor population ($\chi^2$ trend 157, $P < 0.000$), but a similar trend was not found among the patients ($\chi^2$ 0.93, $P = 0.76$). HCV positivity in patient and donor groups is shown in Table 2. Odds ratio (OR) for anti-HCV positivity was highest in patients below 25 years of age, but was not significant in patients older than 56 years of age as compared with the donor population (Table 2). Among the seropositive NHL cases, six had low-grade and three had high-grade lymphoma. T- and B-cell differentiation was not conducted in this population, but in another study using the same population T-cell phenotype was seen in 4% of the cases.

### DISCUSSION

HCV is both a hepatotropic and a lymphotropic virus and it has been proposed as a possible causative factor in some LPDs as well as liver diseases. HCV-related antigens in infected patients have been found in peripheral B and T lymphocytes, lymph nodes and lymphocytes infiltrating the liver (Lai et al, 1998). The lymphotropism of HCV suggests that this virus may be a trigger factor of the clonal B-cell proliferation including essential mixed cryoglobulinaemia and malignant lymphoma (Zignego et al, 1992; Ferri et al, 1993)

The association between HCV infection and various LPDs has been investigated in numerous studies. HCV-related lymphoma has been reported from Italy, Japan, Israel and the USA (Izumi et al,

**Table 1** Anti-HCV positivity in patient groups

| Disease | Number | Age range | Mean age | HCV (+) | % |
|---|---|---|---|---|---|
| NHL | 98 | 16–75 | 45±15 | 9 | 9.18 |
| CLL | 38 | 30–72 | 57±13 | 4 | 10.52 |
| MM | 47 | 33–85 | 60±10 | 5 | 10.63 |
| HD | 36 | 15–70 | 45±13 | 7 | 19.44 |
| ALL | 9 | 16–30 | 19±5 | 1 | 11.10 |
| Total | 228 | 15–85 | 51±16 | 26 | 11.40 |

NHL, non-Hodgkin's lymphoma; CLL, chronic lymphocytic leukaemia; MM, multiple myeloma; HD, Hodgkin's disease.

1996; Sikuler et al, 1997; Zuckerman et al, 1997). The most prominent association between HCV and LPDs has been shown in essential mixed cryoglobulinaemia and Waldenstrom macroglobulinaemia. The prevalence of anti-HCV and/or HCV–RNA positivity has been reported as between 42 and 100% (Agnello et al, 1992; Santini et al, 1993; Ferri et al, 1993; Cacoub et al, 1994; Pozzato et al, 1994; Mussini et al, 1995). In eight studies from Italy, 25% of the patients (range 9–40%) with B-cell NHL were positive for HCV antibodies (Ferri et al, 1994; Cavanna et al, 1995; Mazzuro et al, 1996; Pioltelli et al, 1996; Silvestri et al, 1996; Usto et al, 1996; Luppi et al, 1997; de Rosa et al, 1997). In these studies, HCV positivity was much higher than in age-comparable groups of the Italian population. In the study done by Silvestri, anti-HCV antibodies were detected in the serum of 29 of 311 patients with B-cell NHL. In another study done by Izumi et al (1996), anti-HCV was not detected in any case of non-B-cell NHL and HD, whereas 12 of 54 (22.2%) cases of B-cell NHL were positive for HCV antibody. In three studies from the UK the evidence of HCV infection using anti-HCV antibody and/or HCV–RNA was not detected (Brind et al, 1996; Hanley et al, 1996; McColl et al, 1996). In a study done by Musto et al (1996) the prevalence of HCV as evaluated by both serological and/or molecular analysis in a large group of LPDs, was found to be higher in 150 patients with MM compared to the controls (12.6 vs 5.4%). However, this difference disappeared in patients over 50 years of age. We also found that anti-HCV was not higher in patients older than 56. Furthermore, in the study by Musto et al (1996), the prevalence of anti-HCV was significantly higher than in controls and was independent of age; we observed similar findings in our patients. It would be more reliable and valuable if we could confirm our results with HCV–RNA using polymerase chain reaction; another study has been planned for this purpose. Our anti-HCV antibody positivity is lower than in the Italian and higher than in the UK populations. There remains a problem with HCV and LPD; despite the epidemiological data about HCV, it has yet to be shown as an aetiological factor of NHL. Reliable and easily performed HCV in-situ detection techniques are, therefore, necessary (Lai et al, 1998).

At present, in some series there is insufficient evidence to conclude that chronic HCV infection increases the risk of the development of NHL, while in others it has been suggested that HCV may play a direct pathogenetic role in some chronic LPDs. The relationship between HCV and NHL remains uncertain in our study. The striking geographical variation in the association between NHL and HCV and pathogenetic role of HCV in lymphoproliferation needs further investigations.

In the present study, OR for anti-HCV in the patients with LPDs as compared to the donor population is 24.29. This suggests that HCV infection may be a causative and/or contributing factor in lymphoproliferation.

### ACKNOWLEDGEMENT

We thank Prof Dr Esmeray Acartürk for her careful revision of the manuscript.

### REFERENCES

Agnello V, Chung RT and Kaplan LM (1992) A role for hepatitis C virus infection in type II cryoglobulinemia. *N Engl J Med* **327**: 1490–1495

Brind AM, Watson JP, Burt A, Kesteven P, Wallis J, Proctor SJ and Bassendine MF (1996) Non-Hodgkin's lymphoma and hepatitis C virus infection. *Leuk Lymphoma* **21**: 127–130

Cacoub P, Fabiani FL, Musset L, Perrin M, Franguel L, Leger M, Huraux JM, Pyette JC and Godeau P (1994) Mixed cryoglobulinemia and hepatitis C virus. *Am J Med* **96**: 124–132

De Rosa G, Gobbo ML, Renzo A, Notaro R, Garofalo S, Grimaldi M, Apuzzo A, Chiurazzi F, Picardi M, Matarazzo M and Rotoli B (1997) High prevalence of hepatitis C virus infection in patients with B-cell lymphoproliferative disorders in Italy. *Am J Hematol* **55**: 77–82

Devita S, Zagonel V, Russo A, Rupolo M, Cannizzaro R, Chiara G, Boiocchi M, Carbone A and Frenseschi S (1998) Hepatitis C virus, non-Hodgkin's lymphomas and hepatocellular carcinoma. *Br J Cancer* **77**: 2032–2035

Ferri C, Monti M, La Civita L, Longombardo G, Greco F, Pasero G, Gentilini P, Bombardieri S and Zignego AL (1993) Infection of peripheral blood mononuclear cells by hepatitis C virus in mixed cryoglobulinemia. *Blood* **82**: 3701–3704

Ferri C, Caracciolo F, Zignego AL, Civita LL, Monti M, Longombardo G, Lombardini F, Greco F, Capochiani E, Mazzoni A, Mazzaro C and Pasero G (1994) Hepatitis C infection in patients with non-Hodgkin's lymphoma. *Br J Haematol* **88**: 392–394

Hanley J, Jarvis L, Simmonds P, Parker A and Ludiam C (1996) HCV and non-Hodgkin's lymphoma. *Lancet* **347**: 1339

Izumi T, Sasaki R, Miura Y and Okamoto H (1996) Primary hepatosplenic lymphoma: association with hepatitis C virus infection. *Blood* **87**: 5380–5381

**Table 2** Anti-HCV positivity and OR in patients and donors by age groups

| | Donors | | | Patients | | | | |
|---|---|---|---|---|---|---|---|---|
| Age | HCV(+) | HCV(–) | % | HCV(+) | HCV(–) | % | OR | (95% CI) |
| < 25 | 30 | 10237 | 0.29 | 4 | 22 | 15.38 | 62.04 | (16.97–206.05) |
| 26–35 | 58 | 19494 | 0.30 | 2 | 21 | 8.69 | 32.01 | (invalid) |
| 36–45 | 50 | 5107 | 0.97 | 3 | 32 | 8.57 | 9.58 | (2.26–34.13) |
| 46–55 | 42 | 1358 | 3.00 | 3 | 30 | 9.09 | 3.23 | (0.75–11.69) |
| > 56 | 12 | 128 | 8.57 | 14 | 97 | 12.61 | 1.54 | (0.64–3.74) |
| Total | 192 | 36226 | 0.53 | 26 | 202 | 11.40 | 24.29 | (15.38–38.10) |

 © 1999 Cancer Research Campaign

Lai R and Weiss RM (1998) Hepatitis C virus and non-Hodgkin's lymphoma. *Am J Clin Pathol* **109**: 508–510

Luppi M, Longo G, Ferrari MG, Ferrara L, Marasca R, Barozzi P, Morselli M, Emilia G and Torelli G (1997) Prevalence of HCV infection and second neoplasms in marginal zone lymphomas. *Br J Haematol* **96**: 873–874

Mazzaro C, Zagonel V, Monfardini S, Tulissi P, Pussini E, Fanni M, Sorio R, Bortolus R, Crovatto M, Santini G, Tiribelli C, Sasso F, Masutti R and Pozzato G (1996) Hepatitis C virus and non-Hodgkin's lymphomas. *Br J Haematol* **94**: 544–550

McColl MD and Trait RC (1996) Hepatitis C virus infection in patients with lymphoproliferative disorders. *Br J Haematol* **92**: 771–772

Mussini C, Ghini M, Mascia MT, Giovanardi P, Zanni G, Lattudo I, Moreali S, Longo G, Ferrari MG and Torelli G (1995) Monoclonal gammopathies and hepatitis C virus infection. *Blood* **85**: 1144–1145

Musto P, Dell'Olio M, Carotenuto M, Mangia A and Andriulli A (1996) Hepatitis C virus infection: a new bridge between hematologists and gastroenterologists? *Blood* **88**: 752–753

Pioltelli P, Zehender G, Monti G, Monteverde A and Galli M (1996) HCV and non-Hodgkin lymphoma. *Lancet* **347**: 624–625

Pozzato G, Mazzaro C, Crovatto M, Modolo ML, Ceselli S, Mazzi G, Sulfaro S, Franzin F, Tulissi P, Moretti M and Santini GF (1994) Low grade malignant lymphoma, hepatitis C virus infection, and mixed cryoglobulinemia. *Blood* **84**: 3047–3053

Santini GF, Crovatto M, Modolo ML, Martelli P, Silvia C, Mazzi G, Franzin F, Moretti M, Tulissi P and Pozzato G (1993) Waldenstrom macroglobulinemia: a role of HCV infection. *Blood* **82**: 2932

Sikuler E, Shnaider A, Zilberman D, Hilzenrat N, Shemer-Avni Y, Neumann L and Buskila D (1997) Hepatitis C virus infection and extrahepatic malignancies. *J Clin Gastroenterol* **24**: 87–89

Silvestri F, Pipan C, Barillari G, Zaja F, Fanin R, Infanti L, Russo D, Ffalasca E, Botta GA and Baccarani M (1996) Prevalence of hepatitis C virus infection in patients with lymphoproliferative disorders. *Blood* **87**: 4296–4301

Zignego AL, Macchia D, Monti M, Thiers V, Mazzetti M, Foschi M, Maggi E, Romagnoni S, Gentilini P and Brechot C (1992) Infection of peripheral mononuclear blood cells by hepatitis C virus. *J Hepatol* **15**: 382–386

Zuckerman E, Zuckerman T, Levine AM, Douer D, Gutekunst K, Mizokami M, Qian DG, Valenkar M, Nathwani BN and Fong TL (1997) Hepatitis C virus infection in patients with B-cell non-Hodgkin's lymphoma. *Ann Intern Med* **127**: 423–428

*© 1999 Cancer Research Campaign*
